# Influence of the Sanitary, Economic, and Social Crisis of COVID-19 on the Emotional State of Dentistry in Galicia (Spain)

**DOI:** 10.3390/ijerph20043088

**Published:** 2023-02-10

**Authors:** María Sofía Rey-Martínez, María Helena Rey-Martínez, Natalia Martínez-Rodríguez, Cristina Meniz-García, José María Suárez-Quintanilla

**Affiliations:** 1 Department of Surgery and Medical-Surgical Specialties, Faculty of Medicine and Dentistry, University of Santiago de Compostela, 15705 A Coruña, Spain; 2 Department of Otolaryngology, Central Hospital of the Red Cross of Madrid, 28003 Madrid, Spain; 3 Department of Dental Clinical Specialties, Faculty of Dentistry, Complutense University of Madrid, 28040 Madrid, Spain

**Keywords:** COVID-19, dentistry, psychological burnout, stress and anxiety, professional activity, economic factors

## Abstract

The main objective of this research was to analyze the economic, social, and emotional repercussions among Galician dentists (Spain) as a consequence of the COVID-19 pandemic. A survey was filled out by 347 professionals. After verifying the survey’s reliability using Cronbach’s alpha = 0.84, the professional activity and emotional state of the participants were assessed based on aspects related to their personal and family data. The economic impact of the pandemic was considerable, and all participants experienced a decrease in income. In total, 72% of the participants considered that working with personal protective equipment (PPE) made their clinical activities difficult, and 60% expressed concern about being infected during their professional practice. Among the professionals, women (*p* = 0.005), and separated, divorced, or single professionals (*p* = 0.003) were the most strongly affected. Separated or divorced professionals were the group that most frequently raised the need to make a radical change in their lives. Finally, it was observed that the emotional consequences varied substantially in the lives of these professionals, mainly among female dentists (*p* = 0.010), separated and divorced men (*p* = 0.000), and those with fewer years of professional practice (*p* = 0.021). The COVID-19 pandemic had an economic impact, due to the decrease in the number of patients and hours of attention, as well as an emotional impact, mostly expressed in the form of sleep disorders and stress. The most vulnerable professionals were women and professionals with fewer years of experience.

## 1. Introduction

The SARS-CoV-2 pandemic has changed the ways of life and social habits throughout much of the world and has generated significant cultural and emotional impacts. The pandemic has also highlighted the fragilities and weaknesses of society on a global scale, as well as its strengths, including the dedication of healthcare personnel, the work of research laboratories and pharmaceutical companies around the world, and the social collaboration in the face of this new situation that is fraught with social and emotional difficulties [1].

Depression and anxiety have increased in most countries, among both healthcare professionals and the general population [2,3]. The loss of loved ones who suffered from the most serious consequences of the disease, isolation, job loss (or fear of job loss), economic precariousness and reduced income, mobility restrictions, teleworking, difficulties of conciliation, and a fear of infection are just some of the many causes of the high emotional impact of the pandemic on the general population, which was exacerbated among patients who already suffered from a mental disorder.

Dentistry is considered one of the healthcare professions with the highest risk of contracting infection from aerosols, since most dental activity generates such suspensions. During the first months of the pandemic, recommendations and work protocols were established and became strictly implemented in dental clinics, with the main objective being to achieve the highest levels of safety for patients and professionals.

In the first stage, coinciding with a declaration of the state of alert in Spain (from March to May 2020), despite dentistry being legally considered an essential activity, only emergency dental care was allowed. With the de-escalation of the pandemic, dental clinics progressively recovered a certain sense of normality, although the fear of contagion and the economic crisis created a lower demand for care, which demanded economic reinforcements in the sector that had already been implemented during previous economic crises.

During this period, close to 30% of Spanish dental clinics requested a temporary employment regulation file (ERTE), and an unquantified number of dentists lost their jobs. Therefore, dentists have had to take on many challenges, and the COVID-19 pandemic has influenced their physical and mental health and impacted their familial relationships and financial status, with the added pressure of radically changing their professional protocols. All of these factors have led many dentists to personally reconsider their profession, which was considered by most of society to be successful, emerging, lucrative, necessary, and respected, but whose foundations have been shaken by the small, but powerful force of a virus.

The psychological consequences of this pandemic among the healthcare population were highlighted by Ornell et al. [4], who, in their 2020 research on a population of 1300 healthcare workers, reported that 50% presented symptoms of depression, 44% presented uncontrolled anxiety, and 34% had problems with falling asleep.

Thus, there is little doubt that the COVID-19 pandemic and the corresponding set of complex measures that were taken have generated a profound psychological impact on the general and healthcare population in the form of anguish, anxiety, fear, depression, insomnia, and a denial of reality [5,6,7,8,9,10].

In this way, as reflected by Sacham et al. [11], psychological distress among dental staff could have long-term effects and, as such, future implications for training.

The existence of the repercussions of this pandemic among dentists in different countries should be complemented with the experiences of others to assess the pandemic’s repercussions in a more global context. For this reason, the contributions of new data (which were, until now, practically non-existent) on the COVID-19 pandemic’s impact among Spanish dentists, not only on their professional development but also on their emotional development, can be relevant. 

The main objective of this research was to analyze the economic, social, and emotional repercussions of the COVID-19 pandemic in this professional group, proposing as a null hypothesis that the pandemic would not produce any impact in these areas.

## 2. Materials and Methods

### 2.1. Studio Design

A descriptive observational study was designed during the months of March and July 2021 using a Forms application questionnaire (Alphabet, Mountain View, CA, USA). This questionnaire was sent electronically. The study was carried out in accordance with the Declaration of Helsinki, and was approved by the Ethics and Research Committee of the College of Dentists and Stomatologists of A Coruña (Approval Code: 22/04CEI-AC) on 22 January 2021.

Anonymous and voluntary participation was requested from the professional associations of dentists of A Coruña, Lugo, and Pontevedra-Ourense, to all professionals from Galicia (Spain) who were, at that time, members of these institutions.

### 2.2. Questionnaire Design

To design the questionnaire, different questionnaires widely used in studies on health, job satisfaction, and quality of professional life were reviewed (Personal Achievement Questionnaire, Maslach Burnout Inventory-MBI-HSS [12], Copenhagen Burnout Questionnaire, CBI [13], and Professional Quality of Life Scale version 5 (ProQOL-5) [14]).

A prior evaluation of the questionnaire was carried out to assess its reliability, validity, sensitivity, and specificity, with the participation of 25 professionals from Galicia region. These professionals fulfilled it during the month of February 2021. They were asked to write down the time they needed to complete it, obtaining an average response time of 11 min and 30 s. In terms of reproducibility, a percentage agreement in the responses of 95.1% and a Kappa of 0.879 (95% CI between 0.856 and 0.902) was obtained. To assess the reliability of the questionnaire, the Cronbach’s alpha statistic was used to quantify the level of reliability of a measurement scale for the unobservable magnitude constructed from *n* observed variables. The alpha value was 0.84730613578847.

### 2.3. Selection of Participants and Sample Size

Out of a total of 2208 registered dental professionals in Galicia, 1243 women and 965 men requested to participate in the survey. We established the following as inclusion criteria: Dentists who worked in their own clinics. Professionals who worked for others or in the public system (SERGAS), and those who left the questionnaire incomplete were excluded from the study.

To calculate the sample size and obtain a representative sample, a confidence interval of 95% was estimated for a single proportion for a finite population of size 2208, extending to 0.050 from the observed proportion for an expected proportion of 0.500, resulting in a value of 327 participants.

After receiving the responses of 462 professionals, 107 contracted or employed dentists and 8 who were working in a public center were excluded, with the final sample including 347 self-employed dentists.

### 2.4. Variable Analysis

The sections of the questionnaire were structured as follows:

1. Personal sociodemographic data: age, gender, members of the family unit, marital status, and sick leave.
2. Data on professional practice: years of professional work, specialties, number of clinics, number of dental teams, number of employees, workforce reduction during the pandemic, weekly working hours and working pattern, number of patients seen weekly, percentage decrease in patients, and decrease in income.
3. Data on the emotional state and COVID-19: composed of 24 items that were answered on a Likert scale with four options, assigned values of 0 (never/never), 1 (sometimes/once a month or less), 2 (quite a few times/once a week), and 3 (always/every day).

### 2.5. Statistical Analysis

The descriptive statistics were analyzed with the R program version 4.1.2, and the data obtained were reflected as response frequencies and percentages and 95% confidence intervals.

To analyze the emotional state, two groups were created according to a Likert scale of the scores of the questionnaire items (scores 1 and 2 = not affected; scores 3 and 4 = if affected), with continuous data expressed in medians with ranges. For the interquartile values, percentages, and 95% confidence intervals, unadjusted logistic regression was applied. A value of *p* < 0.05 was considered statistically significant.

## 3. Results

### 3.1. Analysis of Sociodemographic Data

The participants in this study had a mean age of 48 ± 10.30 years, ranging from 26 to 82 years. Women represented 60.81% of the sample, compared to 39.19% for men.

In total, 72.62% were married or had a stable partner, with the number of family members ranging between two and four. Only 8% of the sample had been out of work in the last six months, despite the serious health problems that affected the entire population at the time that the survey was conducted (Table 1).

### 3.2. Analysis of Professional Practice

**Figure 1:**
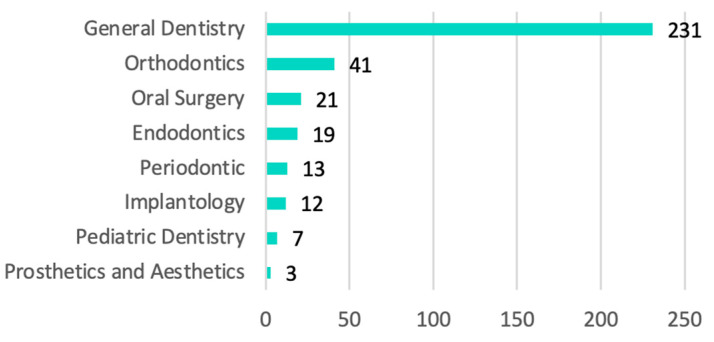
Preferential professional activity.

The average number of years worked up to the moment of carrying out the survey was 20 years, with dedication to general dentistry observed in 66.57% of the professionals, while 33.43% had a preferential dedication to a certain area of dentistry such as orthodontics, surgery, endodontics, periodontics, or oral implantology (Figure 1).

**Figure 2:**
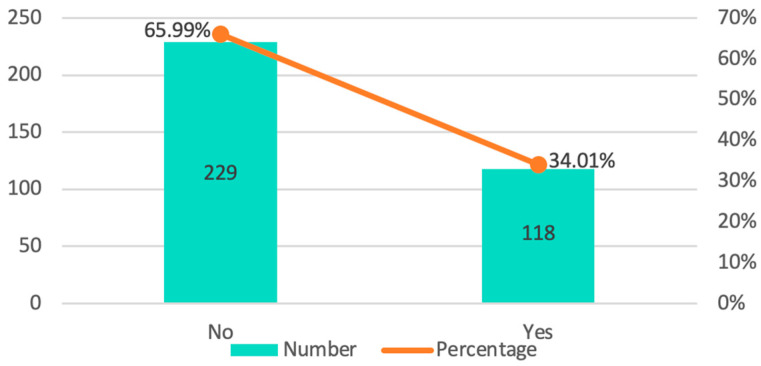
Distribution by frequency and percentages of the clinics as staff was reduced.

Overall, 90.77% worked at between one and three clinics (one clinic: 52.16%; two clinics: 26.51%; and three clinics: 12.1%). In 96.83%, the number of dental chairs ranged between one and five. The number of clinical collaborators prior to the pandemic was mostly between two and five (61.09%), and during the pandemic, 34.01% of those surveyed reported having reduced this workforce (Figure 2).

The working day before the pandemic was much longer than a conventional one. More than half of the professionals (55.62%) worked between 40 and 50 h a week, with only 26.51% performing a continuous shift and 66.86% utilizing two work shifts.

**Figure 3:**
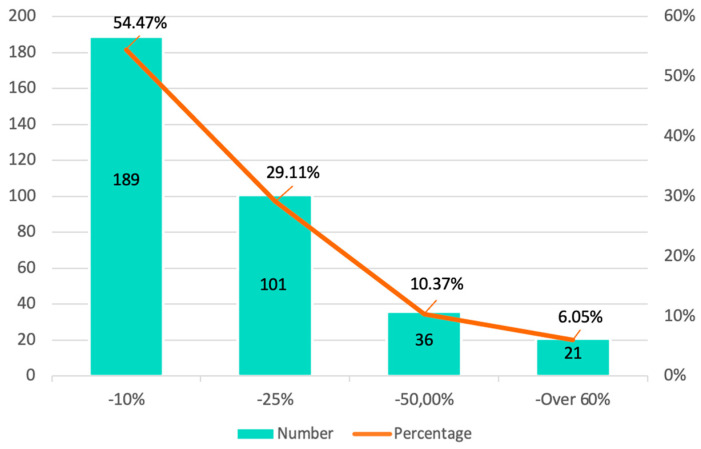
Decrease in the number of patients.

During the pandemic, and especially during the first period of lockdown, there was a general decrease in the number of patients who routinely attended a dental clinic. Thus, approximately 84% of the professionals registered decreases in patients of between 10% and 25%, although among other professionals (10.37%), the decrease during the first phase of the state of alert was close to 50% (Figure 3). 

**Figure 4:**
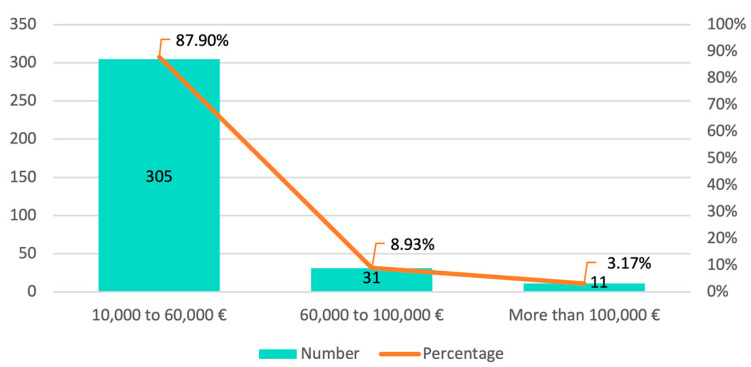
Distribution by frequency and percentages of the decrease in income in dental clinics.

Regarding the economic repercussions of the pandemic on their income, 87.90% of the Galician dental professionals stated that they had experienced a decrease in income of between EUR 10,000 and EUR 60,000 (Figure 4).

### 3.3. Analysis of Emotional State

The analysis of the 24 items is reflected in Table 2, highlighting the following as the most representative results. 

In total, 71% of the professionals considered that they were contributing to improving the pandemic situation, and 72% considered that working with personal protective equipment (PPE) made their clinical activities difficult.

Sixty percent of those surveyed expressed concerns about being infected in their professional practice, and 71% feared being an asymptomatic carrier before vaccination, with female dentists expressing a greater fear of this possibility compared to males (*p* = 0.031). Likewise, the youngest professionals with fewer than 10 years of practice felt greater concern (*p* = 0.026).

Thirty-eight percent of those surveyed stated that they had had insomnia or nightmares; this fear was more significant among female than male dentists (*p* = 0.000). These differences also occurred more frequently among separated, divorced, or single professionals (*p* = 0.000).

A similar response was obtained in relation to feelings of stress or anguish, which was present in 56% of the professionals, with women (*p* = 0.005) and separated, divorced, or single professionals (*p* = 0.003) being those who reported this feeling the most. Moreover, 34% stated that they had cried or experienced signs of despair during the pandemic, with female dentists (*p* = 0.000) and younger professionals (*p* = 0.000) suffering this situation the most frequently.

A feeling of exhaustion at the end of the workday was reported by the majority of the participants, including 65% of professionals, with dentists again reporting the greatest exhaustion (*p* = 0.002).

In relation to the attitudes of the patients, 83% stated that the demands, expectations, and state of mind of patients had changed, and 50% of the professionals responded that they felt more sensitive to the criticism of patients about their treatments; however, no significant differences were found.

In the results of the survey, there was also a clear indication that the pandemic had introduced a new vision of life to many professionals (83%), with this perception being significant among dental professionals (*p* = 0.012).

Fifty-six percent answered that they had felt the need to make a radical change in their lives, with separated or divorced professionals being the group that most frequently considered this circumstance (*p* = 0.003).

Lastly, the questionnaire analyzed whether the emotional lives of these professionals had changed substantially, with 57% of the cases clearly indicating as much. Among this percentage, it was highlighted that the dentists experienced a more intense change in their emotional lives (*p* = 0.010). The same was observed among separated and divorced (*p* = 0.000) participants and among those with fewer years of professional practice (*p* = 0.021).

## 4. Discussion

Dentists had to take on many challenges during the COVID-19 pandemic, which influenced their physical and mental health, family relationships, and financial status, with the added pressure of radically changing their professional protocols.

Most countries have suffered such consequences in a significant way, and very few have experienced a low impact, as indicated by Mekhemar et al. [15] in their study on the German population, who justified this scant impact on the information received by professionals from their government and the provision of financial support.

Gender appears to be an important factor in the psychological consequences [16,17,18,19]. In the present research, it was observed that female dentists were most strongly affected by emotional changes during the pandemic, which is in agreement with other authors, who reported that the causes of this greater affectation could be related to a fear of infecting one’s family members and children, the existence of dependents, or living with relatives with risk factors; these are all elements that could significantly increase manifestations of stress during work activities.

Another relevant aspect is that the greatest number of emotional manifestations were reported among the youngest professionals. These results are in line with those observed by Huang et al. [20], León-Manco et al. [21], and Martina et al. [22] Explanations for this finding could be that dentists who are younger or have briefer work tenures may have expressed a greater fear of losing their jobs due to their shorter work histories, their relative financial insecurity, and their stronger concerns about their career development compared to their more experienced and older peers.

However, other authors such as Dosil et al. [18] found that older healthcare professionals presented greater emotional disturbances, arguing that having dependents, parents, and children would increase their responsibilities and the fear of bringing the virus home. One of the main reasons that could justify this disparity in the results is the different public and private work environments of the professionals, leading to different perceptions of the workplace.

Sleep disorders generally affect a large sector of the population, as corroborated in studies by Iorga et al. [23] (about dentists) and Coico-Lama et al. [24], who observed that 60.1% of medical students presented insomnia.

A lower percentage was observed in this research, again corresponding to the dentists who presented the highest percentages of these disorders. Marital status could be a determining factor, since divorced or separated professionals reported greater sleep disturbances than those with a stable partner. This result could be interpreted as a lack of coexistence with other family members, leading to isolation becoming a trigger.

Another aspect evaluated in this research was the modifications that were made to the participants’ professional activities during the COVID-19 pandemic.

In dental clinics, activity protocols had to be drastically modified, incorporating individual protection measures, adapting the number of working hours, and even reducing the number of collaborators and auxiliary personnel.

The use of PPE influenced aspects such as a concern for its scarcity, the need for greater economic investments, and the difficulties in communicating with patients, leading to emotional repercussions.

Galician dentists mostly stated that the use of PPE had made it more difficult for them to carry out their daily activities. These findings are in line with those observed by Malandkar et al. [25], who reported that 85% of participants agreed that using PPE affected their work efficiency and that 89% experienced communication difficulties.

Another aspect related to PPE is the difficulty in supply that occurred in most of the countries. This circumstance resulted in some professionals considering the possibility of leaving their profession, as reflected by Pacutova et al. [26], Cole et al. [27], and Vick [28]. These findings contrast with those in this study, which determined that “loss of interest in one’s profession or the possibility of leaving it” represented a minority response among those surveyed. Such findings could be explained by the fact that the number of dental professionals in Galicia is not very high relative to other Spanish regions, and the supply of PPE was adequate. Likewise, Galicia had more time to organize itself, since the first wave arrived later than in other regions of Spain and affected fewer people, so there was more information on the impact of COVID-19.

The increase in the number of working hours, together with a drastic modification of patient care protocols, especially those maintained over time, are factors to consider in the increase in anxiety and stress, which can worsen interpersonal relationships [29]. Logically, the transformation of the protocols in dental clinics was carried out in a short span of time, without the existence of evidence-based regulations, along with explicit pressure from patients who demanded security, despite professional teams having little time to adapt to the new reality.

Another unavoidable consequence of the COVID-19 pandemic has been the impact on the financial status of professionals as a result of a decrease in their activities, with fewer daily appointments and greater investments to adapt to the new clinical environment with new preventive protocols, which has affected the emotional state of said professionals [30,31,32].

Tysiąc-Miśta et al. [33], in a study on Polish dentists, highlighted the negative impact of the pandemic on the economy, as did Shinde et al. [34], who, like the majority of Indian dentists, reported a considerable impact on financial concerns, with both emotional and professional repercussions.

A previous study by Chamorro-Petronacci et al. [35], despite being very similar to ours in the number of dental professionals and represented ages, did not reflect the economic reality that appears in this work, and provided very different figures. The authors found that almost 60% of professionals had experienced a drop in income that they estimated to be between EUR 1000 and EUR 10,000, figures that are far from the 87.9% of our surveyed professionals, who estimated a drop in income between EUR 10,000 and EUR 60,000. It is evident that this difference in financial losses could be justified by the fact that the expenses in dental offices increased progressively throughout the successive waves of the virus, clearly decreasing profitability.

Finally, it should be noted that the COVID-19 pandemic, with its corresponding social isolation, has led, in some cases, to individual exclusion, affecting not only professionals but also the patients of our population, generating situations of psychosocial stress [36,37].

Likewise, the stigmatization toward healthcare professionals has been a worrisome situation worldwide for years and has only increased during this pandemic, both verbally and physically [38,39].

As noted by Ghareeb et al. [40], such violence can be caused by colleagues or patients, and the finding in their study demonstrate that the most prevalent type of verbal violence was shouting (90.48%) and threats of harm (58.57%), while shoving (91.67%) and beatings (80.83%) were the predominant types of physical violence. 

Specific research on dentists, such as a study by Rhoades et al. [41], indicates that most members of this professional group have experienced aggression at some point in their career, in the form of physical (45.5%), verbal (74%), and/or reputational (68.7%) attacks. 

Bitencourt et al. [42] recently identified the predictive factors of stigmatization among Brazilian healthcare workers, noting that 47.6% of such workers reported situations of violence during the pandemic and that the most relevant and significant risk factors were not having children or a partner, having fewer than 20 years in the profession, and working more than 36 h per week. 

Physical attacks were also reported, but it was demonstrated that professional criticism from patients had much more negative repercussions than other types of attacks, with the youngest professionals being the most likely to experience this situation.

In terms of the limitations of this study, it has been carried out in the region of Galicia, and there could be variations in relation to other Spanish regions, due to the fact that the economic and professional conditions may be different. Likewise, recall bias in questionnaire responses could be a limiting factor; nevertheless, the average age of the respondents, the social level, and the time of the survey should be sufficient data to minimize this bias.

Lastly, it remains unquestionable whether the COVID-19 pandemic has affected the mental health of a significant proportion of healthcare professionals, including dentists. Therefore, the null hypothesis raised in this research can be rejected. These impacts may be somewhat different between countries, but they converge in a reduction of the resilience of these professionals. 

The present study, like others, should serve to better prepare the healthcare sector for future pandemics, and, as noted by Mira et al. [43], they should sensitize government institutions to support all healthcare more effectively. It is undeniable that the dental profession is subjected, under usual conditions, to a certain degree of stress so it would be advisable to carry out future research to determine those professional or personal aspects that may be more influential in the triggering of emotional disorders. This would allow these professionals to prepare for possible adverse events.

## 5. Conclusions

The COVID-19 pandemic in Galicia (Spain), as in other countries, has had a professional and emotional impact on dentists. The establishment of new prevention measures and protocols caused an economic impact on their professional activities due to the decrease in both the number of patients and hours of attention. 

Emotional impairment was mostly expressed in the form of stress and sleep disorders, with women and younger professionals in this study being those who reported that they were suffering the greatest consequences. 

These lived experiences should help health authorities and dentists to act more effectively in future situations similar to COVID-19.

## Figures and Tables

**Table 1:** Sociodemographic data.

**Gender**	**No. Cases**	**%**
Male	136	39.19
Female	211	60.81
**Marital Status**	**No. Cases**	**%**
Married/Stable partner	252	72.62
Separated/Divorced	43	12.39
Single	45	12.97
Widowed	4	1.15
Other	3	0.87
**Number of Family Members**	**No. Cases**	**%**
1	39	11.24
2	86	24.78
3	75	21.61
4	104	29.97
5	40	11.53
6	3	0.87
**Out of Work in the Last 6 Months**	**No. Cases**	**%**
Yes	28	8.07
No	319	91.93

**Table 2:** Questionnaire on the emotional state.

	Questions	Average	SD	Answer			
				0	1	2	3
1	Do you feel you are helping to improve the current situation of the pandemic?	2.09	1.03	10%	20%	22%	49%
2	Do you think you can catch COVID-19 by carrying out your work as a dentist?	1.86	1.03	9%	31%	24%	36%
3	Have you considered leaving your job to protect yourself and your family?	0.48	0.80	67%	21%	8%	4%
4	Have you felt disappointed in your dentistry work due to the COVID-19 conditions?	1.42	0.93	18%	35%	31%	15%
5	Has PPE made it more difficult to carry out your daily work?	2.16	1.01	9%	19%	26%	46%
6	Have you felt discriminated against as a health professional during the pandemic?	1.33	1.08	28%	29%	24%	19%
7	Is your family scared that you would return home infected with COVID-19?	1.24	1.06	31%	31%	23%	16%
8	Do you live with any family members who are in a high-risk group for COVID-19?	1.20	1.31	49%	13%	11%	27%
9	Were you scared of being an asymptomatic carrier before you were vaccinated?	1.74	1.11	19%	21%	27%	34%
10	Have you had nightmares or insomnia due to COVID-19?	1.16	1.09	38%	24%	24%	14%
11	Do you feel stressed or distressed due to COVID-19?	1.69	1.04	16%	27%	30%	26%
12	Do you feel unmotivated to go to the clinic daily?	1.18	0.95	27%	38%	23%	12%
13	Do you feel emotionally exhausted at the end of the working day?	1.91	0.95	10%	26%	33%	32%
14	Do you think the expectations, demands, and state of mind of patients have changed?	2.26	0.75	2%	15%	41%	42%
15	Do you feel more sensitive to the criticism that patients make about their treatment?	1.52	0.97	17%	33%	33%	17%
16	Have you felt lonely in the face of the management of the pandemic?	1.68	1.07	18%	30%	25%	28%
17	Have you lost interest in dentistry since the pandemic began?	0.87	0.95	44%	31%	17%	8%
18	Have you considered temporarily moving away from the profession?	0.57	0.87	62%	20%	12%	6%
19	Do you feel supported by the rest of your team regarding the measures (change in working hours, change in contracts, furlough, etc.) that you have had to take to deal with the pandemic and its financial consequences?	2.18	0.97	9%	15%	29%	47%
20	Has the pandemic provided you with a new perspective on life?	2.25	0.85	5%	13%	35%	48%
21	Would you wish to make serious changes to the management of protocols and to the management of human resources?	1.63	0.96	12%	31%	34%	23%
22	Have you cried or have you lost hope any day during the pandemic?	1.01	0.95	36%	30%	26%	8%
23	At any time have you felt the need to make a radical change to your life?	1.56	1.08	23%	22%	33%	23%
24	Has your emotional life seen a significant change during the pandemic?	1.65	1.00	14%	28%	32%	25%

SD: standard deviation and PPE: personal protective equipment.

## Data Availability

Data will be provided on request.
